# Kitlg dysfunction is associated with hearing and pigmentation abnormalities

**DOI:** 10.1016/j.gendis.2025.101890

**Published:** 2025-10-22

**Authors:** Yun Xiao, Ruifeng Qiao, Jing Cai, Lei Chen, Yu Jin, Aizhen Zhang, Shuhui Kong, Yongdong Song, Haibo Wang, Lei Xu

**Affiliations:** a Department of Otolaryngology-Head and Neck Surgery, Shandong Provincial ENT Hospital, Cheeloo College of Medicine, Shandong University, Jinan, Shandong 250022, China; b Shandong Institute of Otorhinolaryngology, Jinan, Shandong 250022, China

Kitlg, a pivotal protein involved in neural crest cell migration and pigmentation, harbors pathogenic variants that can lead to non-syndromic hearing loss, Waardenburg syndrome type 2, or familial progressive hyperpigmentation with or without hypopigmentation.^1^ Distinct *KITLG* mutations are associated with diverse clinical outcomes, as evidenced by the phenotypic spectrum presented in Figure S1. Not all patients with *KITLG* variants present with pigmentation abnormalities and hearing loss simultaneously. KITLG is known to bind to its receptor, c-Kit, activating the RAS/MAPK signaling pathway, which regulates the transcription of microphthalmia-associated transcription factor (MITF), a critical transcription factor for melanocyte generation, differentiation, and survival.^2^ To elucidate the mechanisms underlying hearing loss associated with *Kitlg* dysfunction, we generated a mouse model with a heterozygous *Kitlg* mutation (c.81_84 del, p.E27DfsX5) using CRISPR/Cas9 technology (Fig. 1A and B). This mutation induced a frameshift and introduced a premature stop codon. Western blotting and immunofluorescence analyses demonstrated the marked decrease in KITLG protein expression levels in heterozygous *Kitlg*^Δ/+^ mice compared with wild-type (WT) controls (Fig. 1C–E). Most *Kitlg*^*Δ/+*^ mice developed abnormal hair color, such as white hair on the belly and/or forehead (Fig. 1F; Fig. S2A). Some *Kitlg*^*Δ/+*^ mice showed unilateral or asymmetric hearing loss (Fig. 1G; Fig. S2B). The mouse model aligns with clinical phenotypes of patients with *KITLG* mutation, including asymmetric or unilateral deafness and dyspigmentation.^1^

**Figure 1:**
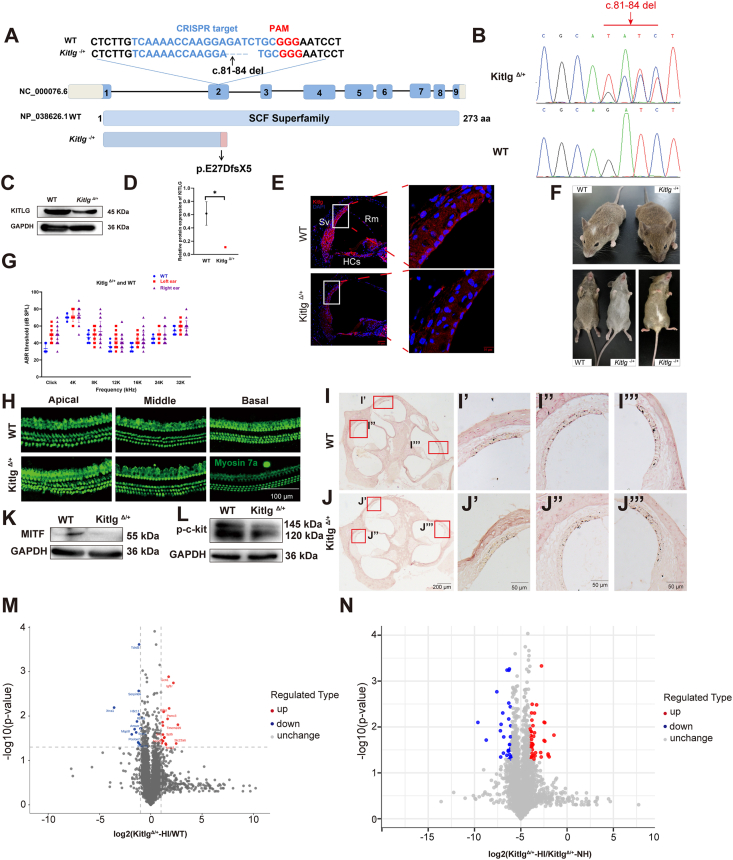
Generation of *Kitlg*^*Δ*/*+*^ mice and the mechanism of hearing loss. **(A)** Schematic for *Kitlg* knockout in mice. **(B)** Representative DNA sequence chromatograms of *Kitlg* knockout heterozygote mice. **(C, D)** Analysis and quantification of Kitlg protein levels. **(E)** Immunofluorescence staining of cochlear cryosections from 4-week-old WT mice revealed Kitlg expression (red) throughout the cochlea. Scale bar = 50 or 10 μm. **(F)** Abnormal hair color was observed in *Kitlg*^*Δ/+*^ mice. **(G)** Auditory brainstem response (ABR) results in mice (*n* = 5 for WT, *n* = 27 for *Kitlg*^*Δ/+*^). **(H)** Images of cochlear hair cells in WT and *Kitlg*^*Δ/+*^ mice at 4 weeks. Scale bar = 100 μm. **(I–I‴)** Masson–Fontana stain analysis revealed the distribution of melanin in the stria vascularis of the 4-week-old WT mouse cochlea. **(J–J‴)** Masson–Fontana stain analysis revealed the distribution of melanin in the stria vascularis of *Kitlg*^*Δ/+*^ mouse cochlea. Scale bar = 200 or 50 μm. **(K, L)** Analysis and quantification of MITF and p-c-kit protein levels. **(M)** The volcano plot shows the significance and fold change of different proteins in the *Kitlg*^*Δ/+*^-HI group versus the WT group. **(N)** The volcano plot shows the significance and fold change of different proteins in the *Kitlg*^*Δ/+*^-HI group versus the *Kitlg*^*Δ/+*^-NH group. Data were presented as mean ± standard error of the mean. ∗*P* < 0.05, ∗∗*P* < 0.01, and ∗∗∗*P* < 0.001, using two-tailed, unpaired Student's *t*-tests.

Using this mouse model, we further investigated the mechanism of hearing loss caused by the *Kitlg* dysfunction. We used immunofluorescence to reveal the subcellular localization of Kitlg in the cochlea of 4-week-old mice, and noted widespread expression within key cochlear components, such as hair cells, stria vascularis, and spiral ganglion neurons (Fig. 1E). Further evaluation of the cochlear basement membrane in 4-week-old mice stained with anti-Myosin7a antibody to label hair cells (green) (Fig. 1H; Fig. S3A) and ribbon synapses of inner hair cells labeled with anti-CtBP2 antibodies revealed comparable numbers of hair cells and CtBP2-positive puncta in *Kitlg*^*Δ/+*^ and WT mice (Fig. S3B). Hematoxylin-eosin staining was used to observe similarities in the overall morphology of the cochlea between *Kitlg*^*Δ/+*^ and WT mice (Fig. S3C and S3D), indicating no difference in the thickness or morphology of the stria vascularis. These findings suggest that *Kitlg* dysfunction does not affect the cochlear structure.

Fontana–Masson staining revealed that *Kitlg*^*Δ/+*^ mice with impaired hearing had reduced melanin distribution in the stria vascularis compared with WT mice (Fig. 1I and J). The transmission electron microscopy analysis revealed that intermediate cells were present in the stria vascularis of *Kitlg*^*Δ/+*^, but exhibited abnormal morphological characteristics compared with the WT littermates (Fig. S4). These findings indicate that KITLG haploinsufficiency may not affect intermediate cell migration, proliferation, or differentiation, but could disrupt their functional maturation, such as melanin synthesis. Steel et al have demonstrated that c-kit (the receptor of KITLG) homozygous mutant mice (W^v^/W^v^) lack intermediate melanocytes in the stria vascularis, leading to loss of the endocochlear potential and profound hearing loss.^3^ This phenotypic divergence likely arises from fundamental differences in the genotypic models employed: whereas c-kit homozygous mutants exhibit complete developmental failure of intermediate melanocytes, partial KITLG deficiency permits their migration but severely compromises functional maturation.

Considering the crucial role of Kitlg/c-Kit signaling pathway in the melanin synthesis, we evaluated the expression of MITF and p-c-kit in *Kitlg*^*Δ/+*^ and WT mice and showed that MITF (Fig. 1K; Fig. S5A) and p-c-kit protein levels (Fig. 1L; Fig. S5B) in *Kitlg*^*Δ/+*^ mice were lower than those in WT mice. To further elucidate the function of *Kitlg* in the auditory system, we employed proteome and phosphoproteome technologies to analyze protein expression (Table S1) and phosphorylation levels (Table S2) in *Kitlg*^*Δ/+*^-HI (*Kitlg*^*Δ/+*^ mice with impaired hearing), *Kitlg*^*Δ/+*^-NH (*Kitlg*^*Δ/+*^ mice with normal hearing), and WT mice. By comparing *Kitlg*^Δ/+^-HI mice with WT mice, as well as *Kitlg*^*Δ/+*^-HI with *Kitlg*^*Δ/+*^-NH genotypes, we successfully identified a significant number of differentially expressed proteins (Fig. 1M and N) and phosphorylated proteins (Fig. S6B and S6C). Comparative proteomic and phosphoproteomic analyses between *Kitlg*^*Δ/+*^-HI and WT groups revealed that the MAPK signaling pathway, identified through KEGG pathway enrichment analysis of differentially phosphorylated proteins, regulates MITF, a key transcription factor governing pigmentation (Fig. S6D). This finding aligns with the known role of Kitlg in activating and modulating the Ras/MAPK signaling pathway, a critical downstream cascade associated with melanogenesis.

We further investigated the compensation mechanism that part of *Kitlg*^*Δ/+*^ mice presented with normal hearing. Notably, the Kyoto Encyclopedia of Genes and Genomes (KEGG) analysis for differentially phosphorylated proteins indicated a high enrichment of some signaling pathways, such as Rap1 and cAMP signaling pathways (Fig. S6E). The cAMP signaling pathway was the initial signal transduction pathway during the formation of avian auditory epithelium to substitute for hair cells.^4^ The cAMP pathway regulates the MAPK pathway both positively and negatively. Therefore, we hypothesize that the substantial enrichment of the cAMP pathway in some *Kitlg*^Δ/+^ mice compensates for the abnormalities in the MAPK pathway caused by dysfunction of Kitlg, thereby preventing hearing loss.

To delineate the functional dynamics of Kitlg during inner ear development, we conducted comparative proteomic and phosphoproteomic profiling of *Kitlg*^Δ/+^ and WT mice at two critical developmental stages: embryonic day 14.5 and postnatal day 2. This temporal analysis revealed stage-specific phosphorylation patterns in the RAS/MAPK and PI3K–Akt pathways, demonstrating Kitlg's dual regulatory roles in early melanocyte survival and late-stage ion homeostasis establishment (Fig. S6F and S6G). These findings, corresponding to the distinct phosphorylated proteins in *Kitlg*^*Δ/+*^-HI and WT mice at 4 weeks, suggested a probable association with auditory dysfunction during development. Specifically, we compared the difference in protein expression between *Kitlg*^Δ/+^ and WT mice at embryonic day 14.5 and postnatal day 2, and also screened numerous hearing-related genes, such as FXYD6, KARS1, and SLC26A2 (Fig. S6H). Notably, FXYD6 plays a crucial role in maintaining endolymph in the cochlea by regulating Na^+^/K^+^-ATPase function.^5^ In the present study, we observed significant reductions in FXYD6 levels (Fig. S6I and S6J). Melanocytes in the stria vascularis serve as intermediate cells, and the functionality of potassium channels in these cells, along with that of Na^+^/K^+^-ATPase, is crucial for maintaining endocochlear potential. FXYD6 down-regulation impairs Na^+^/K + -ATPase activity, exacerbating endocochlear potential loss. This dual-hit model may expand the traditional melanocyte-centric view of Kitlg-associated hearing loss.

In this study, we generated a Kitlg heterozygous mutation mouse model (c.81_84 del, p.E27DfsX5) that recapitulates key clinical features of human KITLG-related disorders, including asymmetric hearing loss and pigmentation anomalies. While cochlear morphology remained intact, *Kitlg*^*Δ/+*^ mice exhibited reduced melanin deposition in the stria vascularis, suggesting that KITLG haploinsufficiency disrupts the functional maturation of intermediate cells, particularly melanin synthesis, without impairing their migration, proliferation, or differentiation. Advanced techniques, such as single-cell RNA sequencing, could provide definitive quantification of intermediate cells and further clarify their role in the pathogenesis of these disorders. Proteomic and phosphoproteomic analyses further revealed impaired RAS/MAPK signaling and down-regulation of MITF, a critical regulator of melanogenesis. Intriguingly, compensatory activation of the cAMP pathway was observed in a subset of *Kitlg*^*Δ/+*^ mice, potentially mitigating hearing loss. Additionally, diminished FXYD6 expression implicated a dual pathogenic mechanism involving both Na^+^/K^+^-ATPase dysfunction and melanocyte-dependent endocochlear potential loss. However, future investigations should delineate the spatiotemporal interplay between compensatory pathways and developmental processes. These findings advance our understanding of KITLG-associated hearing loss, highlighting potential therapeutic targets to restore melanocyte function or modulate compensatory signaling pathways.

## CRediT authorship contribution statement

**Yun Xiao:** Data curation, Writing – original draft, Methodology. **Ruifeng Qiao:** Writing – original draft. **Jing Cai:** Methodology. **Lei Chen:** Methodology. **Yu Jin:** Data curation. **Aizhen Zhang:** Methodology. **Shuhui Kong:** Data curation. **Yongdong Song:** Methodology. **Haibo Wang:** Writing – review & editing, Supervision, Funding acquisition. **Lei Xu:** Writing – review & editing, Supervision, Project administration.

## Ethics declaration

All animal procedures were performed according to the protocols approved by the Animal Care Committee of Shandong University, China (No. ECAESDUSM 20,123,011), consistent with the National Institute of Health's Guide for the Care and Use of Laboratory Animals. Consent to participate was not required.

## Data availability

The datasets used and/or analyzed during the current study are available from the corresponding authors upon reasonable request.

## Funding

This work was supported by the 10.13039/501100001809National Natural Science Foundation of China (No. 32200484, 82271172, 82101225) and the 10.13039/501100007129Natural Science Foundation of Shandong, China (No. ZR2021QH321).

## Conflict of interests

The authors declared no competing interests.
