# Supplementary material for: Kitlg dysfunction is associated with hearing and pigmentation abnormalities
Source: Genes Dis. 2025 Oct 22;13(3):101890. doi: 10.1016/j.gendis.2025.101890 (PMC12824913; doi:10.1016/j.gendis.2025.101890)
Supplement: Multimedia component 1 [file mmc1.docx]

**Supplementary Data**

## 1. Materials and Methods

## Generation of *the Kitlg^Δ/+^* mouse model

*Kitlg^Δ/+^* knockout mice were constructed using CRISPR-Cas9 technology on a CBA mouse background. Specific single guide RNAs (sgRNAs) targeting the regions of exon2 within the Kitlg genes were designed using online tools (http://crispor.tefor.net/). Once the gRNAs were designed, the Cas9 ribonucleoprotein (RNP) complex was assembled. The RNP complex was microinjected into the cytoplasm of fertilized mouse zygotes. After implantation and the birth of the pups, successful mutations were screened using genotyping methods such as PCR amplification followed by Sanger sequencing. Genotyping and sequencing were conducted using the primers F:5’- TGGCGAGAGCAGCTTGAATG-3’, and R: F:5’- GTGTCGGGAGTGCCCTAAAT-3’.

All animal procedures were performed according to the protocols approved by the Animal Care Committee of Shandong University, China (No. ECAESDUSM 20,123,011), consistent with the National Institute of Health’s Guide for the Care and Use of Laboratory Animals. Efforts were made to minimize animal usage and prevent suffering.

## Auditory brainstem response (ABR)

The ABR test, a crucial method for evaluating auditory function in animals, was conducted after anesthetizing mice in a soundproof room. Specifically, each animal was anesthetized with ketamine (50 mg/kg) and xylazine (6 mg/kg) by intramuscular injection and placed on a heating pad (37 °C) (FHC; USA) in a soundproof chamber.

The mice were sterilized with 75% alcohol, the active electrode was placed at the vertex between the eyes, the ground electrode was placed beneath the ear, and the reference electrode was placed at the rear toward the tail of the anesthetized mice. Tucker-Davis Technologies System workstations and software (BioSig and SigGen) provided acoustic waves and assessed responses. The animals were stimulated with click and tone-burst stimuli (4, 8, 12, 16, 24, and 32 kHz). The sound stimulus intensity was decreased from 90 to 20 dB, and the intensity was reduced by 5 dB each time. The auditory threshold had the lowest sound intensity, which could be recorded using a standard waveform.

## Western blotting

Mice were euthanized by cervical dislocation; their cochleae were dissected and homogenized in RIPA lysis buffer (Beyotime, P0013B) containing phenylmethylsulfonyl fluoride. The tissue homogenates were then lysed on ice for 30 min and centrifuged at 10 000 g for 20 min at 4 ℃. The samples were treated with 6× loading buffer, heated to 100 °C for 10 min, and 30 μg of each protein sample was resolved using SDS-PAGE. The samples were then transferred to PVDF membranes (Millipore, IPVH00010), and the membranes were blocked for 1 h at 37 ℃ with 5% skim milk in TBST before incubating with primary antibodies overnight at 4 ℃. After washing with TBST, the membranes were incubated for 1 h at room temperature with a suitable HRP-conjugated secondary antibody. After washing with TBST, the bands were visualized using an ECL reagent (Yamei). The following primary antibodies were used: SCF (1:1000, Proteintech, 26582-1-AP), p-c-kit (1:1000, Cell Signaling Technology, 3391S), FXYD6 (1:1 000, Santa Cruz Biotechnology, sc-398465), MITF (1:1 000, Proteintech, 13092-1-AP), GAPDH (mouse polyclonal, Proteintech, 60004-1-Ig). Semi-quantification of the western blot results was performed using ImageJ software to measure the intensities of the bands. Each experiment was repeated at least three times.

## Histological analysis

Following euthanasia by cervical dislocation, cochlea was fixed in 4% paraformaldehyde (4 °C overnight), decalcified in 0.5 mM EDTA (room temperature, 24 h), dehydrated in a sucrose gradient (10%, 15%, 30%, and 30% sucrose: OCT, Solarbio 1:1), embedded in OCT, and stored at -80 °C until further use. The specimen was then sectioned to 5-µm thickness, and the frozen sections were either stained with H&E or Masson–Fontana Melanin Stain Kit (Solarbio, Beijing). Finally, the specimen was visualized using a light microscope (Nexcope, NIB910).

## Immunofluorescence analysis

Frozen cochlea sections were removed, incubated at room temperature (20–30 ℃) for 10 min, and soaked in methanol for 10 min. Specimens were washed thrice with phosphate-buffered saline (PBS) and soaked in a quick antigen retrieval solution (Solarbio) for 30 min at room temperature. Sections were blocked with 10% goat serum at 37 °C for 60 min. Subsequently, the sections were incubated with primary antibodies at 4 °C overnight, washed thrice with PBS, and incubated with secondary antibodies at room temperature for 60 min. Finally, a DAPI fluorescence mounting medium (Abcam, ab104139) was used to mount the specimen, and images were acquired using a laser scanning confocal microscope (Leica SP8). The following antibodies were used: Myosin-Ⅶa (Rabbit Polyclonal Antibody, 1:400, Proteus-Biosciences, 25-6790), CtBP2 (Mouse, 1:200, BD Biosciences, 612044), and SCF (rabbit polyclonal, 1:1 000, Abcam, ab52603).

## Transmission electron microscopy (TEM)

For observation of distribution of melanocytes in the SV, the cochlea of WT and *Kitlg ^Δ/+^* mice were fixed in 1% glutaraldehyde at 4 ℃. The SV of cochlea was separated under a microscope and fixed for 2 h at 4 ℃ in 1% tetroxide, washed in PBS, dehydrated in graded ethanol, embedded in Epon 812 resin, and eventual visualization under the electron.

## Proteomic and phosphoproteomic analysis

Cochleae were collected on E14.5 and P2, and at 4 weeks of age in mice and immediately placed on dry ice. Subsequently, these samples were sent to Shanghai Applied Protein Technology Co., Ltd. for mass spectrometric analyses, and the spectral data provided by the company were meticulously analyzed.Liquid Chromatography - Tandems Mass Spectrometry analysis was performed using a timsTOF Pro mass spectrometer (Bruker) coupled to Nanoelute (Bruker). The raw MS data for each sample were combined and analyzed using MaxQuant 1.6.14 software to identify and quantify proteins. Cluster 3.0 (http://bonsai.hgc.jp/~mdehoon/software/cluster/software.htm) and Java Treeview software (http://jtreeview.sourceforge.net) were employed for hierarchical clustering analysis. The Euclidean distance algorithm for similarity measurement and the average linkage clustering algorithm, utilizing centroids of the observations, were selected for hierarchical clustering. Additionally, a heat map was presented alongside a dendrogram for data visualization. DEPs, modified peptides, genes, and metabolites were mapped to pathways using the KEGG; http://www.kegg.jp/). Enrichment analysis was performed, and the KEGG annotation and enrichment results of the two omics datasets were combined using R (Version 3.5.1). Venn diagrams and bar plots were generated.

## Statistical analysis

All data are expressed as mean ± SEM, and each experiment was repeated at least thrice to ensure the validity and reproducibility of our results. GraphPad Prism 8 software was used for statistical analyses. We employed two-tailed unpaired Student’s t-tests to assess the statistical significance between the two groups. P-values < 0.05 were deemed statistically significant, with significance levels denoted as *p < 0.05, **p < 0.01, and ***p < 0.001.

2. **Supplementary Figures**

**Figure S1.** Schematic illustration of the *KITLG* gene showing the positions of all reported mutations associated with FPHH (green), WS (red), or hearing loss (blue).

**Figure S2. Abnormal pigmentation and auditory dysfunction in *Kitlg^Δ/+^* mice.** (A) Proportion of *Kitlg^Δ/+^* mice with different abnormal coat colors (n = 101). (B) Auditory brainstem response (ABR) results in mice with asymmetric hearing loss (n =5).

**Figure S3. Normal cochlear structure in *Kitlg^Δ/+^* mice.** (A) Quantification of the number of hair cells (n = 3). (B) Staining of ribbon synapse structures in the cochlea of WT and *Kitlg^Δ/+^* mice. Synapses were stained with the presynaptic anti‐CtBP2 (green). Scale bar = 10 µm. (C–D’’) H&E staining analysis of the 4-week-old WT (C, C’, C’’) and *Kitlg^Δ/+^* mice cochleae (D, D’, D’’) showed no significant differences in morphology of the Reissner’s membrane (Rm), stria vascularis (Sv), tectorial membrane (Tm), and organ of Corti. Scale bar = 200 µm, 50 µm, 20 µm.

**Figure S4.** The transmission electron microscopy for stria vascularis of *Kitlg ^Δ/+^* and WT mice. LC, light intermediate cells. DC, dark intermediate cells. *, blood vessel. Scale bar = 5 µm.

**Figure S5.** (A) Analysis and quantifications of MITF protein levels. (B) Analysis and quantifications of p-c-kit protein levels. Data are presented as mean ± SEM *p < 0.05, **p < 0.01, ***p < 0.001, using two-tailed, unpaired Student's t-tests.

**Figure S6. Proteome and phosphorylated proteome comparison in cochlea of *Kitlg^Δ/+^* -HI*, Kitlg^Δ/+^* -NH and WT mice.** (A) Quantitative statistics results for Venn of quantified proteins. (B) Volcano plot showing the significance and fold change of different phosphorylated proteins in group *Kitlg^Δ/+^*-HI vs. WT. (C) Volcano plot showing the significance and fold change of different phosphorylated proteins in group *Kitlg^Δ/+^*-HI vs. *Kitlg^Δ/+^*-NH. (D) KEGG annotated bar chart with differentially phosphorylated proteins in *Kitlg^Δ/+^*-HI vs. WT group. (E) KEGG annotated bar chart with differentially phosphorylated proteins in *Kitlg^Δ/+^*-HI vs. *Kitlg^Δ/+^*-NH group. (F and G) KEGG annotated bar chart with differentially expressed phosphorylated proteins in groups WT (P2 vs. E14.5) and Kitlg^Δ/+^ (P2 vs. E14.5), respectively. (H) Significance and fold change of hearing-related proteins in *Kitlg^Δ/+^* and WT mice during the developmental stages, E14.5 and P2. (I and J) Analysis and quantifications of FXYD6 protein levels. Data are presented as mean ± SEM *p < 0.05, **p < 0.01, ***p < 0.001, using two-tailed, unpaired Student's t-tests.
